# Supplementary material for: Personality links with lifespan in chimpanzees
Source: eLife. 2018 Oct 9;7:e33781. doi: 10.7554/eLife.33781 (PMC6177254; doi:10.7554/eLife.33781)
Supplement: Supplementary file 1. [file elife-33781-supp1.docx]

Personality links with lifespan in chimpanzees

Supplementary File 1

Containing:

Tables S1 – S4

**Table S1.**

| **Accelerated failure time survival models of rearing conditions, origin, and sex.** *N* = 194 (107 deaths). Both models used the Weibull error distribution. | | | | | | | | |  |
| --- | --- | --- | --- | --- | --- | --- | --- | --- | --- |
|  | Without sex | | | |  | Including sex | | | |
|  | Estimate | 95% | C.I. | Hazard Ratio |  | Estimate | 95% | C.I. | Hazard Ratio |
| Nursery-reared | 0.00 | [-0.11, | 0.12] | 0.97 |  | 0.03 | [-0.08, | 0.18] | 0.97 |
| Wild-born | -0.05 | [-0.32, | 0.23] | 1.17 |  | -0.08 | [-0.33, | 0.14] | 1.32 |
| Sex |  |  |  |  |  | -0.25 | [-0.35, | -0.14] | 2.46 |
|  | | | | |  |  |  |  |  |

Table S2.

**Survival model estimates of personality and demographic variables related to longevity.** Estimates and confidence intervals are computed as weighted averages from the set of models in Table S3.

|  |  | Unadjusted | | |  | Adjusted for age | | |
| --- | --- | --- | --- | --- | --- | --- | --- | --- |
| Variable |  | Hazard Ratio | 95% | C.I. |  | Hazard Ratio | 95% | C.I. |
| Sex |  | 1.58 | [1.15, | 2.19] |  | 1.61 | [1.16, | 2.22] |
| Wild-born | | 1.28 | [0.89, | 1.84] |  | 1.28 | [0.89, | 1.84] |
| Agreeableness | | 0.84 | [0.68, | 1.04] |  | 0.84 | [0.64, | 1.01] |
| Dominance | | 1.07 | [0.90, | 1.27] |  | 1.10 | [0.91, | 1.33] |
| Extraversion | | 1.16 | [0.91, | 1.49] |  | 1.11 | [0.83, | 1.49] |
| Conscientiousness | | 1.10 | [0.89, | 1.36] |  | 1.14 | [0.89, | 1.47] |
| Neuroticism | | 0.99 | [0.83, | 1.18] |  | 1.00 | [0.82, | 1.21] |
| Openness | | 0.86 | [0.70, | 1.04] |  | 0.87 | [0.73, | 1.04] |
|  |  |  |  |  |  |  |  |  |

Table S3.

**Individual regression estimates and information criteria for whole sample models used in weighted averages.** Agr = agreeableness, Dom = dominance, Ext = extraversion, Con = conscientiousness, Neu = neuroticism, Opn = openness, K = numbers of parameters in the model, AIC = Akaike Information Criterion. Weightings for wild-born and sex estimates do not include all models, thus their weights differ slightly but not substantially, and are not shown. As a binary variable, a 1 for sex indicates a male.

|  | Wild-born | Sex | Agr | Dom | Ext | Con | Neu | Opn | *K* | *ΔAIC* | Weight |
| --- | --- | --- | --- | --- | --- | --- | --- | --- | --- | --- | --- |
| Unadjusted |  |  |  |  |  |  |  |  |  |  |  |
| Weibull | 0.32 | 0.48 | -0.17 | 0.01 | 0.17 | 0.10 | -0.02 | -0.13 | 11 | 7.61 | 0.01 |
|  | 0.35 |  | -0.19 | 0.06 | 0.24 | 0.06 | -0.01 | -0.19 | 10 | 8.18 | 0.00 |
|  |  | 0.46 | -0.16 | 0.06 | 0.13 | 0.12 | 0.00 | -0.13 | 10 | 3.61 | 0.08 |
|  |  |  | -0.16 | 0.11 | 0.19 | 0.09 | 0.01 | -0.19 | 9 | 9.39 | 0.00 |
| Splines | 0.26 | 0.46 | -0.19 | 0.05 | 0.11 | 0.09 | -0.03 | -0.12 | 23 | 17.36 | 0.00 |
|  | 0.32 |  | -0.19 | 0.10 | 0.17 | 0.06 | -0.02 | -0.19 | 22 | 23.16 | 0.00 |
|  |  | 0.49 | -0.17 | 0.07 | 0.10 | 0.10 | -0.03 | -0.12 | 22 | 17.06 | 0.00 |
|  |  |  | -0.17 | 0.12 | 0.17 | 0.06 | -0.02 | -0.18 | 21 | 23.78 | 0.00 |
| Gompertz | 0.24 | 0.45 | -0.19 | 0.06 | 0.16 | 0.09 | -0.01 | -0.16 | 11 | 0.36 | 0.39 |
|  | 0.29 |  | -0.19 | 0.10 | 0.21 | 0.06 | 0.00 | -0.22 | 10 | 5.73 | 0.03 |
|  |  | 0.47 | -0.17 | 0.08 | 0.14 | 0.10 | -0.01 | -0.15 | 10 | 0.00 | 0.46 |
|  |  |  | -0.17 | 0.12 | 0.17 | 0.06 | 0.00 | -0.21 | 9 | 6.10 | 0.02 |
| Log-logistic | 0.42 | 0.48 | -0.10 | 0.08 | 0.00 | 0.18 | 0.06 | -0.13 | 11 | 26.88 | 0.00 |
|  | 0.46 |  | -0.09 | 0.13 | 0.03 | 0.14 | 0.07 | -0.20 | 10 | 33.01 | 0.00 |
|  |  | 0.50 | -0.06 | 0.12 | -0.05 | 0.20 | 0.08 | -0.13 | 10 | 29.52 | 0.00 |
|  |  |  | -0.04 | 0.18 | -0.03 | 0.17 | 0.10 | -0.19 | 9 | 36.49 | 0.00 |
| Adjusted |  |  |  |  |  |  |  |  |  |  |  |
| Weibull | 0.31 | 0.48 | -0.18 | 0.05 | 0.10 | 0.13 | -0.01 | -0.12 | 11 | 7.03 | 0.02 |
|  | 0.34 |  | -0.20 | 0.11 | 0.18 | 0.09 | 0.00 | -0.19 | 10 | 13.57 | 0.00 |
|  |  | 0.49 | -0.17 | 0.07 | 0.10 | 0.13 | -0.01 | -0.11 | 10 | 7.87 | 0.01 |
|  |  |  | -0.18 | 0.15 | 0.14 | 0.11 | 0.01 | -0.17 | 9 | 8.27 | 0.01 |
| Splines | 0.26 | 0.47 | -0.19 | 0.09 | 0.03 | 0.14 | -0.02 | -0.10 | 23 | 16.04 | 0.00 |
|  | 0.31 |  | -0.20 | 0.14 | 0.10 | 0.10 | -0.01 | -0.16 | 22 | 22.19 | 0.00 |
|  |  | 0.50 | -0.18 | 0.11 | 0.03 | 0.14 | -0.02 | -0.10 | 22 | 15.76 | 0.00 |
|  |  |  | -0.18 | 0.16 | 0.10 | 0.10 | -0.01 | -0.15 | 21 | 22.79 | 0.00 |
| Gompertz | 0.24 | 0.47 | -0.18 | 0.08 | 0.10 | 0.13 | 0.00 | -0.13 | 11 | 0.37 | 0.42 |
|  | 0.28 |  | -0.19 | 0.13 | 0.17 | 0.10 | 0.01 | -0.19 | 10 | 6.25 | 0.02 |
|  |  | 0.48 | -0.17 | 0.10 | 0.10 | 0.13 | -0.01 | -0.13 | 10 | 0.00 | 0.51 |
|  |  |  | -0.18 | 0.15 | 0.18 | 0.09 | 0.00 | -0.18 | 9 | 6.56 | 0.02 |
| Log-logistic | 0.45 | 0.45 | -0.15 | 0.12 | -0.02 | 0.21 | 0.06 | -0.12 | 11 | 25.18 | 0.00 |
|  | 0.49 |  | -0.16 | 0.18 | 0.02 | 0.17 | 0.08 | -0.17 | 10 | 30.52 | 0.00 |
|  |  | 0.48 | -0.12 | 0.16 | -0.06 | 0.22 | 0.09 | -0.12 | 10 | 28.90 | 0.00 |
|  |  |  | -0.11 | 0.22 | -0.03 | 0.18 | 0.11 | -0.17 | 9 | 35.12 | 0.00 |

Table S4.

**Individual regression estimates and information criteria for sex split models used in weighted averages.** Agr = agreeableness, Dom = dominance, Ext = extraversion, Con = conscientiousness, Neu = neuroticism, Opn = openness, K = numbers of parameters in the model, AIC = Akaike Information Criterion. Weightings for wild-born and sex estimates do not include all models, thus their weights differ slightly but not substantially, and are not shown. As a binary variable, a 1 for sex indicates a male.

|  | Wild-born | Agr | Dom | Ext | Con | Neu | Opn | *K* | *ΔAIC* | Weight |
| --- | --- | --- | --- | --- | --- | --- | --- | --- | --- | --- |
| Male |  |  |  |  |  |  |  |  |  |  |
| Unadjusted |  |  |  |  |  |  |  |  |  |  |
| Weibull | 0.50 | -0.41 | -0.10 | 0.07 | 0.09 | -0.12 | 0.09 | 10 | 3.07 | 0.11 |
|  |  | -0.39 | 0.00 | 0.04 | 0.13 | -0.08 | 0.06 | 9 | 2.65 | 0.14 |
| Spline | 0.34 | -0.44 | -0.03 | 0.05 | 0.10 | -0.08 | 0.07 | 22 | 22.53 | 0.00 |
|  |  | -0.42 | 0.03 | 0.04 | 0.13 | -0.07 | 0.05 | 21 | 21.36 | 0.00 |
| Gompertz | 0.26 | -0.43 | -0.04 | 0.04 | 0.10 | -0.10 | 0.10 | 10 | 1.46 | 0.25 |
|  |  | -0.42 | -0.01 | 0.03 | 0.11 | -0.09 | 0.08 | 9 | 0.00 | 0.51 |
| Log-logistic | 0.87 | -0.37 | -0.02 | 0.04 | 0.14 | -0.04 | 0.03 | 10 | 15.82 | 0.00 |
|  |  | -0.29 | 0.16 | 0.00 | 0.22 | 0.06 | -0.06 | 9 | 20.48 | 0.00 |
| Adjusted |  |  |  |  |  |  |  |  |  |  |
| Weibull | 0.43 | -0.49 | -0.08 | 0.05 | 0.15 | -0.10 | 0.06 | 10 | 2.79 | 0.12 |
|  |  | -0.48 | 0.01 | 0.05 | 0.16 | -0.08 | 0.04 | 9 | 1.93 | 0.18 |
| Spline | 0.29 | -0.50 | 0.01 | 0.00 | 0.20 | -0.06 | 0.06 | 22 | 22.64 | 0.00 |
|  |  | -0.49 | 0.05 | 0.00 | 0.20 | -0.05 | 0.04 | 21 | 21.23 | 0.00 |
| Gompertz | 0.22 | -0.49 | -0.02 | 0.00 | 0.19 | -0.07 | 0.07 | 10 | 1.61 | 0.22 |
|  |  | -0.48 | 0.01 | -0.01 | 0.19 | -0.07 | 0.06 | 9 | 0.00 | 0.48 |
| Log-logistic | 0.83 | -0.47 | -0.01 | 0.07 | 0.19 | -0.03 | 0.01 | 10 | 14.71 | 0.00 |
|  |  | -0.41 | 0.15 | 0.07 | 0.23 | 0.06 | -0.07 | 9 | 19.08 | 0.00 |
| Female |  |  |  |  |  |  |  |  |  |  |
| Unadjusted |  |  |  |  |  |  |  |  |  |  |
| Weibull | 0.12 | 0.13 | 0.02 | 0.12 | 0.01 | -0.07 | -0.23 | 10 | 0.98 | 0.24 |
|  |  | 0.12 | 0.03 | 0.12 | 0.02 | -0.07 | -0.24 | 9 | 1.51 | 0.18 |
| Spline | 0.17 | 0.09 | 0.03 | 0.06 | -0.02 | -0.11 | -0.22 | 22 | 19.30 | 0.00 |
|  |  | 0.11 | 0.00 | 0.05 | -0.02 | -0.12 | -0.21 | 21 | 17.75 | 0.00 |
| Gompertz | 0.18 | 0.09 | 0.05 | 0.17 | 0.00 | -0.08 | -0.29 | 10 | 1.45 | 0.19 |
|  |  | 0.10 | 0.05 | 0.15 | 0.00 | -0.08 | -0.28 | 9 | 0.00 | 0.39 |
| Log-logistic | 0.15 | 0.19 | 0.04 | -0.11 | 0.05 | -0.04 | -0.23 | 10 | 12.87 | 0.00 |
|  |  | 0.21 | 0.04 | -0.12 | 0.05 | -0.04 | -0.22 | 9 | 11.34 | 0.00 |
| Adjusted |  |  |  |  |  |  |  |  |  |  |
| Weibull | 0.14 | 0.21 | 0.05 | -0.01 | -0.01 | -0.07 | -0.19 | 10 | 1.67 | 0.17 |
|  |  | 0.23 | 0.05 | -0.01 | -0.02 | -0.07 | -0.18 | 9 | 0.00 | 0.39 |
| Spline | 0.18 | 0.18 | 0.05 | -0.06 | -0.02 | -0.11 | -0.18 | 22 | 18.83 | 0.00 |
|  |  | 0.19 | 0.05 | -0.07 | -0.03 | -0.12 | -0.17 | 21 | 17.32 | 0.00 |
| Gompertz | 0.18 | 0.19 | 0.05 | 0.06 | -0.02 | -0.08 | -0.22 | 10 | 2.01 | 0.14 |
|  |  | 0.21 | 0.05 | 0.06 | -0.02 | -0.09 | -0.21 | 9 | 0.56 | 0.30 |
| Log-logistic | 0.26 | 0.22 | 0.09 | -0.22 | 0.03 | -0.02 | -0.19 | 10 | 0.00 | 0.00 |
|  |  | 0.24 | 0.08 | -0.22 | 0.03 | -0.03 | -0.17 | 9 | 0.00 | 0.00 |
